# Predicting in-hospital mortality from Coronavirus Disease 2019: A simple validated app for clinical use

**DOI:** 10.1371/journal.pone.0245281

**Published:** 2021-01-14

**Authors:** Bianca Magro, Valentina Zuccaro, Luca Novelli, Lorenzo Zileri, Ciro Celsa, Federico Raimondi, Mauro Gori, Giulia Cammà, Salvatore Battaglia, Vincenzo Giuseppe Genova, Laura Paris, Matteo Tacelli, Francesco Antonio Mancarella, Marco Enea, Massimo Attanasio, Michele Senni, Fabiano Di Marco, Luca Ferdinando Lorini, Stefano Fagiuoli, Raffaele Bruno, Calogero Cammà, Antonio Gasbarrini

**Affiliations:** 1 Gastroenterology Hepatology and Transplantation, ASST Papa Giovanni XXIII–Bergamo, Bergamo, Italy; 2 Department of Infectious Diseases, IRCCS Fondazione Policlinico San Matteo, Pavia, Italy; 3 Pneumology Unit, ASST Papa Giovanni XXIII–Bergamo, Bergamo, Italy; 4 UOC di Medicina Interna e Gastroenterologia, Dipartimento di Scienze Gastroenterologiche, Endocrino-Metaboliche e Nefro-Urologiche, Fondazione Policlinico Universitario A. Gemelli IRCCS, Rome, Italy; 5 Section of Gastroenterology and Hepatology, Department of Health Promotion, Mother and Child Care, Internal Medicine and Medical Specialties, University of Palermo, Palermo, Italy; 6 Department of Surgical, Oncological and Oral Sciences (Di.Chir.On.S.), University of Palermo, Palermo, Italy; 7 Cardiovascular Department and Cardiology 1 Unit, ASST Papa Giovanni XXIII–Bergamo, Bergamo, Italy; 8 Department of Economics, Business and Statistics (SEAS), University of Palermo, Palermo, Italy; 9 Hematology and Bone Marrow Transplant Unit, ASST Papa Giovanni XXIII–Bergamo, Bergamo, Italy; 10 Department of Health Promotion, Mother and Child Care, Internal Medicine and Medical Specialties, University of Palermo, Palermo, Italy; 11 Emergency and Intensive care Department, ASST Papa Giovanni XXIII–Bergamo, Bergamo, Italy; 12 Department of Clinical, Surgical, Diagnostic and Pediatric Sciences, University of Pavia, Pavia, Italy; National Institute for Infectious Diseases Lazzaro Spallanzani-IRCCS, ITALY

## Abstract

**Backgrounds:**

Validated tools for predicting individual in-hospital mortality of COVID-19 are lacking. We aimed to develop and to validate a simple clinical prediction rule for early identification of in-hospital mortality of patients with COVID-19.

**Methods and findings:**

We enrolled 2191 consecutive hospitalized patients with COVID-19 from three Italian dedicated units (derivation cohort: 1810 consecutive patients from Bergamo and Pavia units; validation cohort: 381 consecutive patients from Rome unit). The outcome was in-hospital mortality. Fine and Gray competing risks multivariate model (with discharge as a competing event) was used to develop a prediction rule for in-hospital mortality. Discrimination and calibration were assessed by the area under the receiver operating characteristic curve (AUC) and by Brier score in both the derivation and validation cohorts. Seven variables were independent risk factors for in-hospital mortality: age (Hazard Ratio [HR] 1.08, 95% Confidence Interval [CI] 1.07–1.09), male sex (HR 1.62, 95%CI 1.30–2.00), duration of symptoms before hospital admission <10 days (HR 1.72, 95%CI 1.39–2.12), diabetes (HR 1.21, 95%CI 1.02–1.45), coronary heart disease (HR 1.40 95% CI 1.09–1.80), chronic liver disease (HR 1.78, 95%CI 1.16–2.72), and lactate dehydrogenase levels at admission (HR 1.0003, 95%CI 1.0002–1.0005). The AUC was 0.822 (95%CI 0.722–0.922) in the derivation cohort and 0.820 (95%CI 0.724–0.920) in the validation cohort with good calibration. The prediction rule is freely available as a web-app (COVID-CALC: https://sites.google.com/community.unipa.it/covid-19riskpredictions/c19-rp).

**Conclusions:**

A validated simple clinical prediction rule can promptly and accurately assess the risk for in-hospital mortality, improving triage and the management of patients with COVID-19.

## Introduction

Severe acute respiratory syndrome coronavirus 12 (SARS-CoV-2) was first identified in China in December 2019 and has since spread rapidly all over the world [1]. Outside China, Italy was the first western country to be involved and the first case was diagnosed on February, 21. During the initial weeks of the pandemic, the rapid increase in cases overwhelmed the capacity of the National Health System to receive and manage patients and to respond in terms of availability of health resources [2]. In the context of triaging patients in emergency departments or in special clinics set up during an acute outbreak, the lack of clinical criteria to identify the most severe cases and to define the evolution of the disease has made the management of the pandemic even more difficult.

A risk stratification of COVID-19 patients is crucial in order to improve the healthcare organization and to best manage a new potential second wave of the epidemic in the coming winter. In this complex epidemiological and clinical scenario, a competing risks model is a robust statistical method to predict patients risk profile when more than one competing event, such as in-hospital mortality and discharge, is present [3]. The aims of this retrospective multicenter study are 1) to derive a simple clinical prediction rule capable of promptly identifying risk factors for in-hospital mortality and discharge in hospitalized patients with COVID-19 by competing risks analysis; 2) to validate this prediction rule in an external validation cohort; 3) to design a free web-app for calculating the risk of in-hospital mortality (COVID-CALC).

## Materials and methods

### Sources of data and definition of variables

We analyzed an integrated database that contained clinical, laboratory and treatment data from all hospitalized patients with a diagnosis of COVID-19 at three Italian referral tertiary centers, two in Lombardy (the “eye of the SARS-COV-2 storm” in Italy) (Bergamo and Pavia) and one in Lazio (Rome): 1) Hospital Papa Giovanni XXIII, Bergamo, Lombardy; 2) Fondazione IRCCS Policlinico San Matteo, Pavia, Lombardy; 3) Fondazione Policlinico Univerisitario A. Gemelli IRCCS, Rome, Lazio. All consecutive patients admitted between February 22^nd^ and April 7^th^, 2020 were enrolled and were followed up until April 30^th^, 2020.

Information on the history and physical examination of hospitalized patients with COVID-19 were abstracted from chart reviews by medical officers at each hospital. Variables collected through standardized recording forms included age, sex, comorbidities, smoking status, time of symptoms onset and time of hospital admission. Additional variables were the presence of fever (defined as axillary temperature of at least 37.5°C), dyspnoea, cough and diarrhea. Investigations consisted of chest radiography or computed tomography and hematologic and biochemical blood tests, including complete blood count, coagulation profile, glutamic pyruvic transaminase (GPT), lactate dehydrogenase (LDH), C-reactive protein (CRP), and creatine kinase. Arterial-blood gas analysis (ABG) was performed when clinical signs of oxygen impairment were detected (e.g. tachypnoea and hypoxia). P/F ratio was calculated as the ratio between PaO2 and FiO2.

Laboratory confirmation of the SARS COV-2 infection was defined as positive real-time reverse transcriptase polymerase chain reaction (RT-PCR) from nasal and pharyngeal swab; samples were prospectively collected and analyzed at the Molecular Virology Units of each center according to the WHO guidelines and Corman et al. protocols [4, 5]. More details are provided in Supplementary Materials.

Treatments included the use of antiviral therapy (Lopinavir/ritonavir or darunavir/ritonavir), hydroxychloroquine, enoxaparin, and immunomodulatory/immunosuppressive therapy such as corticosteroids, Tocilizumab and Sarilumab. Lopinavir/ritonavir 400/100 mg was administered orally twice daily for 14 days, while Darunavir/ritonavir 800/100 mg was administered once daily for 14 days. Hydroxychloroquine 600 mg was administered twice on day 1, followed by a dose of 400 mg daily for 7 days. Enoxaparin 1 mg/kg was given once or twice daily. Corticosteroid treatment consisted in dexamethasone 20 mg daily for 5 days or methylprednisolone 1 mg/kg intravenously daily for 5 days. Tocilizumab 8 mg/kg was administered intravenously in 1 or 2 doses. A second dose was administered after 8–12 hours from the first dose in patients with inadequate response. Sarilumab 400 mg was administered intravenously once. Oxygen support consisted of low (cannula and simple masks) and high flow (Venturi and reservoir masks, Nasal High Flow), helmet continuous positive airway pressure (CPAP) or non-invasive ventilation (NIV). The choice for the oxygen support was determined by rapid deterioration of P/F ratio and upgraded if a further worsening after two hours of treatment was detected.

The Institutional Review Boards of the three centres (ASST Papa Giovanni XXIII–Bergamo, Italy; IRCCS Fondazione Policlinico San Matteo, Pavia, Italy; Fondazione Policlinico Universitario A. Gemelli IRCCS, Rome, Italy) which comply with the Declaration of Helsinki and its revisions, approved this study. All accessed data were fully anonymized.

### Outcomes

The primary outcome was in-hospital mortality. Discharge was analyzed as a competing event in the competing risks survival analysis. The competing risks model proposed by Fine and Gray was applied [6].

The criteria for discharge were absence of fever, resolution of respiratory symptoms, oxygen saturation higher than 94% and two consecutive nasal swab negative for SARS-CoV-2 obtained at least 24 hours apart.

### Statistical analysis

Data for continuous variables are expressed as mean and standard deviation or median and interquartile ranges (IQR), and data for categorical variables are expressed as frequency and percentage. Differences between continuous data were assessed by Student t test or by Mann-Whitney U test. Differences between categorical variables were assessed by χ^2^ test.

In-hospital mortality and discharge were evaluated by competing risks survival analysis, represented by cumulative incidence function (CIF) [3]. The Fine and Gray proportional sub-distribution hazard model was fitted in order to estimate the effect of covariates on CIFs in-hospital death and discharge [6]. Covariates used for multivariate analyses were chosen based on their significance in the univariate analysis (p<0.10). Covariates in the final model with a p-value <0.05 were considered statistically significant. The results are presented as adjusted hazard ratios (HR) and their 95% confidence intervals (CI). Competing risks analyses were performed in SAS version 9.4. Hotdeck missing imputation data and the assessment of discrimination and calibration and were performed in R Core Team (2019). We used hot.deck function from hot.deck library in R 3.8.0.

### Derivation and validation process

Risk factors for in-hospital mortality and discharge identified by competing risks multivariate analysis in the derivation set were used to generate a prediction rule. The probability of dying or of being discharged within 40 days after hospital admission was computed for a hypothetical patient identified by a combination of prognostic factors. The prediction accuracy of the fitted models was assessed by discrimination and calibration both in the derivation (internal validation) and validation cohorts (external validation) [7]. Discrimination of the models was assessed by the area under the receiver operating characteristic curve (AUC or C-index) [8]. Calibration was evaluated by comparing the predicted probability with the observed probability at a certain time point by a calibration plot. Finally, the Brier score, which takes into account both the discrimination and the calibration at the same time, was also calculated. It is defined as the expected squared distance between the observed status at that time and the predicted probability [9]. Thus, a smaller value of the Brier score indicates a better model.

To assess the internal validity of the prediction rule, the derivation set was randomly split into a training set (70%) and a test set (30%) [10]. The external validation of the prediction rule was carried out with data from an external validation cohort, represented by the Rome unit, in terms of discrimination, calibration and the Brier score.

The prediction rule has been translated into a web-app that is freely available to the public (COVID-CALC: https://sites.google.com/community.unipa.it/covid-19riskpredictions/c19-rp). (S1 Fig).

## Results

From February 22nd to April 7th, 2020, a total of 2191 consecutive confirmed cases of COVID-19 were observed. Baseline characteristics of patients stratified according to derivation (n = 1810) and validation cohort (n = 381) are shown in Table 1. Median age was 67 years (IQR, 56–77 years) and 45% of patients were 70 years or older. Sixty-nine percent of patients were male. In 27.5% of patients, at least one comorbidity was present, with hypertension and diabetes being the most common (43% and 17% of patients, respectively). Median time from symptoms onset to hospital admission was 8 days (IQR 5–11 days). At hospital admission, fever was present in 85%, dyspnoea in 56% and cough in 44% of patients. Lymphocyte count lower than 1000/mmc was observed in 77% of the patients, and platelet count was lower than 150000/mmc in 37.5% of patients. CRP was increased in 83% of patients, and LDH resulted elevated in 88% of patients. In the derivation cohort, male sex, hypertension and obesity were significantly more frequent and the prevalence of chronic kidney disease, chronic obstructive lung disease and malignancies was significantly lower in comparison with the validation set. Patients in the derivation set had higher median GPT, CRP, LDH, and D-dimer levels, higher lymphocyte count and lower P/F ratio, in comparison with patients in the validation set.

**Table 1:** Demographic, clinical and laboratory characteristics of patients with Coronavirus Disease-19 on hospital admission in the derivation and the validations cohorts.

	Overall (n = 2191)	Derivation cohort (n = 1810)	Validation cohort (n = 381)	*p*-value
Age (years)	67 (56–77)	67 (55–78)	68 (57–77)	0.960
<50	293 (13.4%)	239 (13.2%)	54 (14.2%)	
50–59	394 (18.0%)	312 (17.2%)	82 (21.5%)	
60–69	511 (23.3%)	437 (24.1%)	74 (19.4%)	
70–79	594 (27.1%)	510 (28.2%)	84 (22.0%)	
>80	399 (18.2%)	312 (17.2%)	87 (22.8%)	
Male sex	1521 (69.4%)	1280 (70.7%)	241 (63.3%)	0.006
Median duration of symptoms before hospital admission	8 (5–11)	8(2–10)	7(5–11)	<0.0001
Duration of symptoms before hospital admission shorter than 10 days	1473 (67.2%)	1193 (65.9%)	280 (73.5%)	0.004
Fever	1866 (85.2%)	1495 (82.6%)	371 (97.4%)	<0.0001
Dyspnea	1235 (56.4%)	1070 (59.1%)	165 (43.3%)	<0.0001
Cough	969 (44.2%)	742 (41.0%)	227 (59.5%)	<0.0001
Diarrhea	164 (7.5%)	127 (7.0%)	37 (9.7%)	0.810
Number of comorbidities				0.250
0	1506 (71.4%)	1233 (71.3%)	273 (71.7%)	
1	446 (21.1%)	372 (21.5%)	74 (19.4%)	
≥ 2	157 (7.4%)	123 (7.1%)	34 (8.9%)	
Comorbidity				
Hypertension	952 (43.4%)	825 (45.6%)	127 (33.3%)	<0.0001
Diabetes	372 (17.0%)	311 (17.1%)	61 (16.0%)	0.370
Obesity	320 (14.6%)	265 (14.6%)	54 (14.1%)	0.014
Coronary Heart Disease	209 (9.5%)	159 (8.8%)	43 (11.2%)	0.230
Chronic kidney disease	164 (7.5%)	124 (6.8%)	40 (10.5%)	0.047
Chronic obstructive lung disease	148 (6.8%)	102 (5.6%)	46 (12.1%)	0.001
Malignancy	98 (4.5%)	66 (3.6%)	32 (8.4%)	0.002
Chronic Liver disease	45 (2.0%)	42 (2.3%)	3 (0.7%)	0.005
Current smoker	87 (4.0%)	63 (3.5%)	24 (6.3%)	0.011
Glutamic pyruvic transaminase, U/L	54(108.61)	57(120.05)	44(57.04)	0.003
C-reactive protein, mg/dL	11.5 (10.01)	11.9 (10.33)	10.1 (8.68)	0.001
C-reactive protein>10 mg/dL	1438/1733 (83.0%)	1145/1359 (84.3%)	293/374 (78.3%)	0.100
Lactate dehydrogenase, U/L	441 (323)	462 (343)	343 (173)	<0.0001
Lactate dehydrogenase>250 U/L	1931 (88.1%)	1648 (91.0%)	273 (71.7%)	0.007
Creatine kinase, U/L	875 (2288)	2834 (3850)	198 (403)	<0.0001
D-dimer, U/L	7680 (26055)	10059 (13667)	5835 (32479)	0.049
White Cell Blood Count, × 10⁹ per L	7.82 (5.93)	7.94 (4.66)	7.42 (8.94)	0.270
Lymphocyte Count, × 10⁹ per L	1.04 (1.84)	1.12 (2.14)	0.86(0.52)	0.001
Lymphocyte Count<1.0 × 10⁹ per L	1678 (76.6%)	1484 (82.0%)	194 (50.9%)	<0.0001
Platelet Count, × 10⁹ per L	142 (98)	141 (97)	146 (100)	0.410
Platelet Count < 150 × 10⁹ per L	823 (37.5%)	675 (37.3%)	148 (38.8%)	0.480
P/F ratio	238 (117)	203(116)	406 (68)	<0.0001

Data are expressed as mean (standard deviation), median (interquartile range) or n (%).

Data on treatments and outcomes according to derivation and validation cohorts are reported in Table 2. Corticosteroid treatment was administered to 129 patients (11%. Data available in 1164 patients). Enoxaparin was given to 254 patients (21%. Data available in 1218 patients). Hydroxychloroquine was administered to 931 patients (80%. Data available in 1163 patients). Seven-hundred seventy-nine patients (49%) received antiviral treatment with Lopinavir/ritonavir and 242 with Darunavir/ritonavir (15%) (Data available in 1593 patients). Tocilizumab was administered in 112 patients (9%) and Sarilumab in 51 patients (13%) (Data available in 1233 patients). One-hundred sixty-four patients (9%) received non-invasive ventilation (Data available in 1765 patients).

**Table 2:** Treatments and outcomes of patients with Coronavirus Disease-19 in the derivation and the validation cohorts.

	Overall (n = 2191)	Derivation cohort (n = 1810)	Validation cohort (n = 381)	*p*-value
**Treatments**				
Corticosteroids	129/1164 (11.1%)	112/783 (14.3%)	17 (4.5%)	<0.0001
Dexamethasone Methylprednisolone	69/129 (53.5%) 60/129 (46.5%)	60/112 (53.6%) 52/112 (46.5%)	9 (52.9%) 8 (47.1%)	
Enoxaparin	254/1218 (20.9%)	67/837 (8.0%)	187 (49.1%)	<0.0001
Hydroxychloroquine	931/1163 (80.0%)	577/784 (73.6%)	354 (92.9%)	<0.0001
Lopinavir /ritonavir	779/1593 (48.9%)	670/1212 (55.3%)	109 (28.6%)	<0.0001
Darunavir/ritonavir	242/1593 (15.2%)	0/1212 (0)	242 (63.5%)	-
Tocilizumab	112/1233 (9.1%)	31/852 (3.6%)	81 (21.3%)	<0.0001
Sarilumab	51/1233 (4.1%)	0/852 (0)	51 (13.4%)	-
Non-invasive ventilation	164/1765 (9.2%)	108/1384 (7.8%)	56 (14.7%)	0.001
**Outcomes**				
Death	540 (24.6%)	495 (27.3%)	45 (11.8%)	<0.0001
Median time from symptoms onset to death (days)	13 (9–19)	13 (9–18)	12 (7–21)	0.60
Median time from hospital admission to death (days)	5 (3–10)	5 (3–9)	8 (5–15)	0.003
Admission to ICU	302 (13.8%)	242 (13.4%)	60 (15.7%)	0.308
Median time from symptoms onset to ICU admission (days)	11 (8–16)	11 (8–16)	18 (7–14)	0.250
Median time from hospital admission to ICU admission (days)	3.5 (1–6)	3.5 (1–6)	3.5 (1–6)	0.710
Discharge	1358 (62.0%)	1057 (58.4%)	301 (79%)	<0.0001
Median time from symptoms onset to discharge (days)	19 (14–26)	19 (13–25)	21 (16–27)	0.010
Median time from hospital admission to discharge (days)	10 (6–16)	8 (5–15)	14 (10–16)	<0.0001

Data are expressed as median (interquartile range) or n (%).

### Clinical outcomes

At the end of follow-up, 540 patients had died (24.6%), 302 (13.7%) had been transferred to ICU, 1358 patients (62.0%) had been discharged and 258 were still hospitalized. Median time from symptoms onset to death and from hospital admission to death were 13 days (IQR 9–19) and 5 days (IQR 3–10), respectively. Median time from hospital admission to ICU admission was 3.5 days (IQR 1–6). Median time from hospital admission to discharge was 10 days (IQR 6–16).

**Fig 1:**
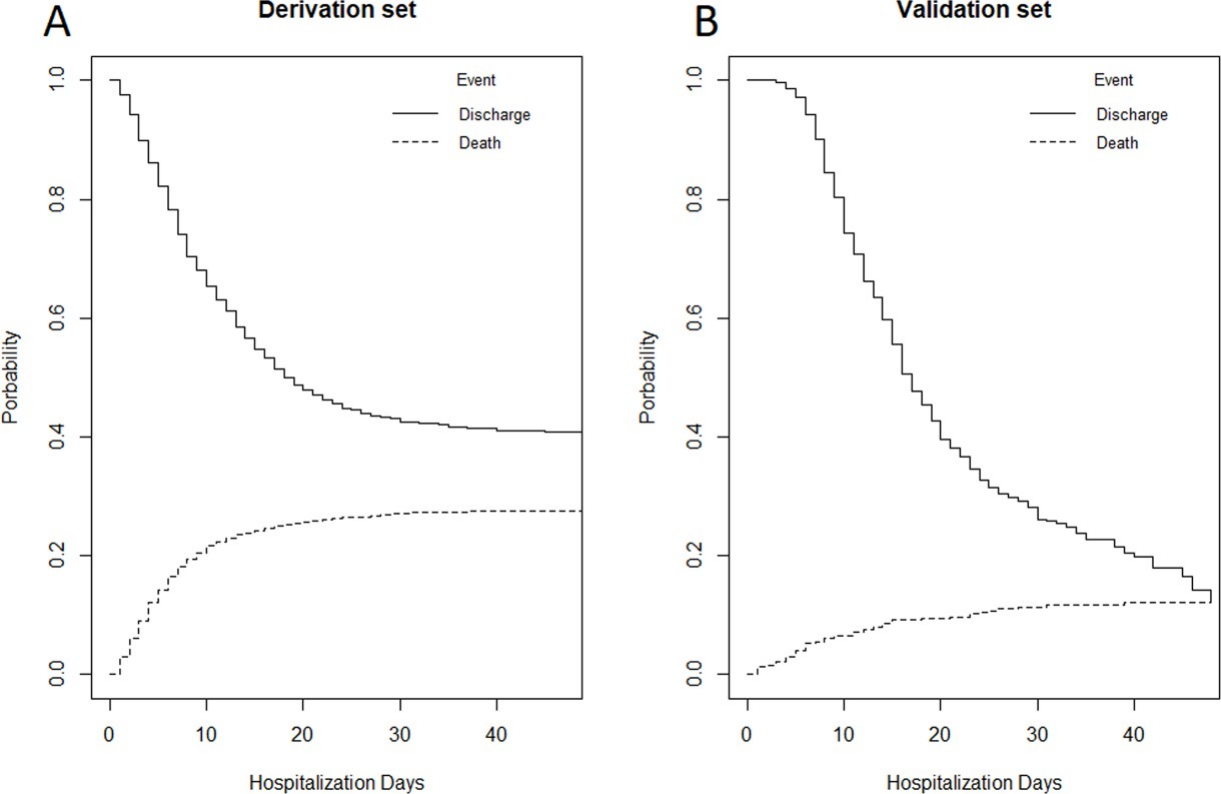
Cumulative incidence functions (CIFs) for in-hospital mortality and discharge of patients with Coronavirus Disease-19 in the derivation (1A) and validation cohorts (1B).

The CIFs for in-hospital mortality and discharge in the derivation and validation cohorts are shown in Fig 1. In-hospital mortality at 7 and 21 days was 16% and 26% in the derivation cohort and 5% and 10% in the validation cohort, respectively. Discharge rates at 7 and 21 days were 22% and 52% in the derivation cohort and 6% and 62% in the validation cohort, respectively.

### Risk factors for in-hospital mortality

Univariate analysis for in-hospital mortality in the derivation cohort is reported in S1 Table in S1 File. In the multivariate model, seven variables were independently associated with in-hospital mortality: age (HR 1.08, 95% CI 1.07–1.09, *p*<0.0001), male sex (HR 1.62, 95% CI 1.30–2.00, *p*<0.0001), duration of symptoms before hospital admission shorter than 10 days (HR 1.72, 95% CI 1.39–2.12, *p*<0.0001), type 2 diabetes (HR 1.21, 95% CI 1.02–1.45, *p* = 0.044), coronary heart disease (HR 1.40, 95% CI 1.09–1.80, *p* = 0.009), chronic liver disease (HR 1.78, 95% CI 1.16–2.72, *p* = 0.008), and LDH levels (HR 1.0003, 95% CI 1.0002–1.0005, *p*<0.0001) (Table 3). Similar results were obtained for discharge model (see S1 Table in S1 File and Table 3). When covariates with p-value <0.20 at univariate analysis were included in the multivariate model, similar results were obtained (S2 Table in S1 File).

**Table 3:** Risk factors for in-hospital mortality and discharge of patients with Coronavirus Disease-19 in the derivation cohort.

	Derivation cohort							
	In-hospital mortality				Discharge			
	Beta	Standard Error	HR (95% CI)	p value	Beta	Standard Error	HR (95% CI)	p value
Age (years)	0.074	0.004	1.08 (1.07–1.09)	<0.0001	-0.027	0.002	0.97 (0.96–0.98)	<0.0001
Male sex	0.481	0.111	1.62 (1.30–2.00)	<0.0001	-0.206	0.068	0.81 (0.71–0.93)	0.002
Duration of symptoms before hospital admission shorter than 10 days	0.542	0.108	1.72 (1.39–2.12)	<0.0001	-0.280	0.063	0.76 (0.67–0.85)	<0.0001
Type 2 diabetes	0.194	0.090	1.21 (1.02–1.45)	0.044	-0.315	0.087	0.73 (0.62–0.86)	0.0003
Coronary heart disease	0.335	0.129	1.40 (1.09–1.80)	0.009	-0.322	0.129	0.72 (0.56–0.93)	0.013
Chronic liver disease	0.576	0.217	1.78 (1.16–2.72)	0.008	-0.613	0.271	0.54 (0.32–0.92)	0.024
Lactate dehydrogenase, U/L	0.0004	0.00008	1.0003 (1.0002–1.0005)	<0.0001	-0.002	0.0002	0.998 (0.997–0.999)	<0.0001

**Fig 2:**
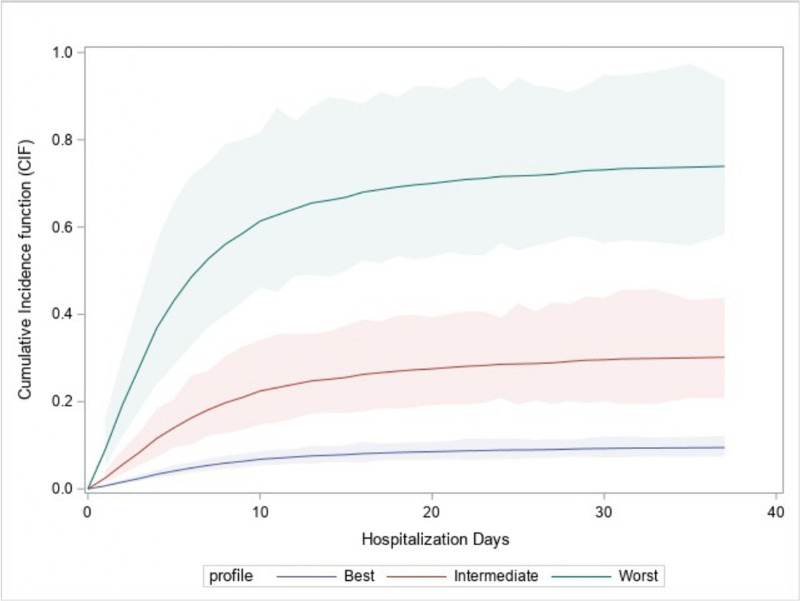
Cumulative Incidence Functions (CIFs) for in-hospital mortality of patients with Coronavirus Disease-19 according to three different patient profiles. A: best profile (60 years old, female sex, duration of symptoms before hospital admission longer than 10 days, no comorbidities, and LDH levels of 250 U/L). B: intermediate profile (70 years old, male sex, duration of symptoms before hospital admission shorter than 10 days, chronic liver disease, LDH levels of 300 U/L.) C: worst profile (80 years old, male sex, duration of symptoms before hospital admission shorter than 10 days, coronary heart disease, chronic liver disease, diabetes, LDH levels of 400 U/L).

These risk factors were used to construct a model encompassing patients grouped into “best”, “intermediate” and “worst” profiles. CIFs for the best (60 years old, female, duration of symptoms before hospital admission longer than 10 days, no comorbidities, and LDH levels of 250 U/L), the intermediate (70 years old, male, duration of symptoms before hospital admission shorter than 10 days, chronic liver disease, LDH levels of 300 U/L) and the worst profiles (80 years old, male, duration of symptoms before hospital admission shorter than 10 days, coronary heart disease, chronic liver disease, diabetes, LDH levels of 400 U/L) are shown in Fig 2. In the best profile, 7- and 21-day in-hospital mortality was 5% and 8%, respectively; in the intermediate profile, 7- and 21-day in-hospital mortality was 18% and 28%, respectively; in the worst profile, 7- and 21-day in-hospital mortality was 52% and 70%, respectively.

### Validation of the prediction rule

By internal validation, the AUC based on the data from the derivation cohort was good (AUC = 0.822, 95% CI 0.722–0.922). The accuracy in the validation cohort was similar to that of the derivation cohort (AUC = 0.820, 95% CI 0.724–0.920). S2 Fig shows the calibration plot of the model for in-hospital mortality. The Brier score was 14.3 in the derivation cohort and 16.9 in the validation cohort. Similar results were obtained for discharge model (see S3 Fig)

The prediction for in-hospital mortality has been translated into a web-based app (COVID-CALC) to obtain both the CIF for in-hospital mortality (predicted curve) and confidence intervals for the CIF at 7, 14 and 21 days (https://sites.google.com/community.unipa.it/covid-19riskpredictions/c19-rp).

## Discussion

In this study, we developed and validated a simple clinical prediction rule able to predict in-hospital mortality of hospitalized patients with COVID-19, considering discharge as a competing risk. In our analysis, seven variables (older age, male sex, shorter duration of symptoms before hospital admission, diabetes, coronary heart disease, chronic liver disease, and LDH levels) were independent risk factors for in-hospital death, as shown by a competing risks multivariate analysis. External validation of this prediction rule showed good discrimination and calibration. To support clinicians in the risk stratification, a web-based app was developed.

From a practical point of view, our prediction rule could help physicians to improve the allocation of medical resources, potentially reducing the overcrowding that we have witnessed in healthcare systems which significantly impacted mortality worldwide during the COVID-19 pandemic. Several prediction models have been previously published aiming to stratify the risk of in-hospital mortality in patients with COVID-19, in both Western and Eastern countries [11–16]. Particularly, the 4C Mortality score [11], including age, sex, number of comorbidities, respiratory rate, oxygen saturation, level of consciousness, urea and CRP levels, was developed in a cohort of more than 35,000 European patients, showing a good discrimination for mortality (AUC = 0.79). Moreover, a 10-item risk score predicting the occurrence of critical illness, defined as a composite of ICU admission, invasive ventilation, or death, was recently validated in a Chinese cohort, showing an AUC of 0.88 [12]. However, our methodological approach was quite different to those used in the above quoted studies. It should be noted that the use of a composite endpoint considers ICU and death to be equal, which may not be true. Moreover, the traditional logistic regression model neglects to model discharge as a competing endpoint. Our competing risks analysis may provide further insights into the effect of clinical covariates on the separate endpoint components [17, 18].

Results of our analyses confirmed those of previous reports from China and the USA [19–21], showing older age as the most important risk factor for in-hospital death in COVID-19. However, we found higher in-hospital mortality in comparison to other studies [19, 20]. The demographic structure of the Italian population could be a reason for this finding. In 2019, Italy resulted as being the European country with the highest proportion of elderly people, with about a quarter of the population aged older than 65 years [22]. Not surprisingly, the median age in our cohort was 67 years, that is higher if compared with that observed in other studies.

Interestingly, in our analysis comorbidities were associated with in-hospital death independently from age and other covariates. In our study, the prevalence of comorbidities was similar to that reported in other Western countries [21], but it was higher when compared to Chinese patients [19, 20], with cardiovascular comorbidities, including coronary heart disease, resulting as the most common. Regarding chronic liver disease, our findings are also in line with the results of two international reporting registries of 152 patients (103 of them with cirrhosis), showing a mortality of about 40% [23].

A shorter duration of the symptoms before hospital admission was independently associated with higher in-hospital mortality. This is a novel finding, and it could be argued that patients with the most severe disease were hospitalized shortly after symptoms onset, while those who were hospitalized after a longer duration of symptoms were those with milder disease.

LDH levels resulted as being independently associated with a higher risk of in-hospital death. LDH is released from cells upon damage of cytoplasmic membrane and its levels might reflect tissue necrosis related to immune hyperactivity, which thus relates to poor outcome [24]. The prognostic role of LDH has also been reported in other Chinese reports [25, 26] and in studies conducted on other coronaviruses [27].

Regarding treatments, it should be underlined that the aim of our analysis was not to assess their efficacy on clinical outcomes of hospitalized patients with COVID-19. Therefore, we described solely the employed treatments and their differences among centers, as the design of our analysis does not allow us to draw firm conclusions regarding the efficacy and safety of the treatments available for COVID-19.

Our study suffers from several limitations. 1) Retrospective studies have many problems that reduce their internal and external validity and selection bias can lead to incorrect results and spurious associations. However, we believe that selection bias could not be relevant as only consecutive patients with COVID-19 were included. 2) A limitation of any prediction rule is the generalizability of results to different populations and settings. However, we performed an external validation that showed good calibration and discrimination. 3) Our derivation and validation cohorts showed significant baseline clinical differences, probably because data were collected in two different settings (Northern vs Central Italy) with different degrees of overcrowding for healthcare systems. However, it should be underlined that hospitalization criteria were similar among participating centres. 4) Patients in our cohort were collected during the early phase of the spread of the infection locally, therefore it may not fit during different epidemic periods. Whether this prediction rule will also apply to patients observed at a later phase of the pandemic remains to be tested. 5) Mortality was limited to in-hospital death, and we assumed discharged patients to still be alive during the study period. 6) The sample size of our validation cohort was relatively small, probably reflecting the differences in disease burden between Northern and Central Italy. 7) The high number of missing data on treatments, particularly regarding corticosteroids use, hampered their inclusion in the prediction rule. However, it should be underlined that the effects of most drug interventions are currently highly uncertain, particularly for the timing of steroids use and the optimal dosage of hydroxychloroquine [28], and no definitive evidence exists that therapies could result in important benefits and harms for any outcomes, as recently reported in a network meta-analysis [29]. 8) LDH was included in our prediction rule, although LDH levels may be not always available. In order to accommodate for possible LDH missingness in the app, we implemented two different prediction rules: the first one based on the final model including LDH values when available, the second one based on a model estimated including all the risk factors of the final model but LDH. Moreover, in the latter case the app will warn the prediction rule is not accurate as the first one.

In conclusion, we developed and validated a simple prediction rule capable of accurately predicting the risk for in-hospital mortality and discharge of patients with COVID-19. Our prediction rule could improve the triage and management of patients with COVID-19 in different epidemiological and healthcare organization settings.

## Supporting information

S1 Checklist (DOCX)

S1 Fig The online web-based calculator for predicting in-hospital mortality among patients with Coronavirus Disease-19. (TIF)

S2 Fig Calibration curves for predicting in-hospital mortality of patients with Coronavirus Disease-19 in the derivation and validation cohorts. (TIF)

S3 Fig Calibration curves for predicting discharge of patients with Coronavirus Disease-19 in the derivation and validation cohorts. (TIF)

S1 File (DOCX)

S1 Dataset (XLSX)
