# Supplementary figures and images for: Predicting in-hospital mortality from Coronavirus Disease 2019: A simple validated app for clinical use
Source: PLoS One. 2021 Jan 14;16(1):e0245281. doi: 10.1371/journal.pone.0245281 (PMC7808616; doi:10.1371/journal.pone.0245281)

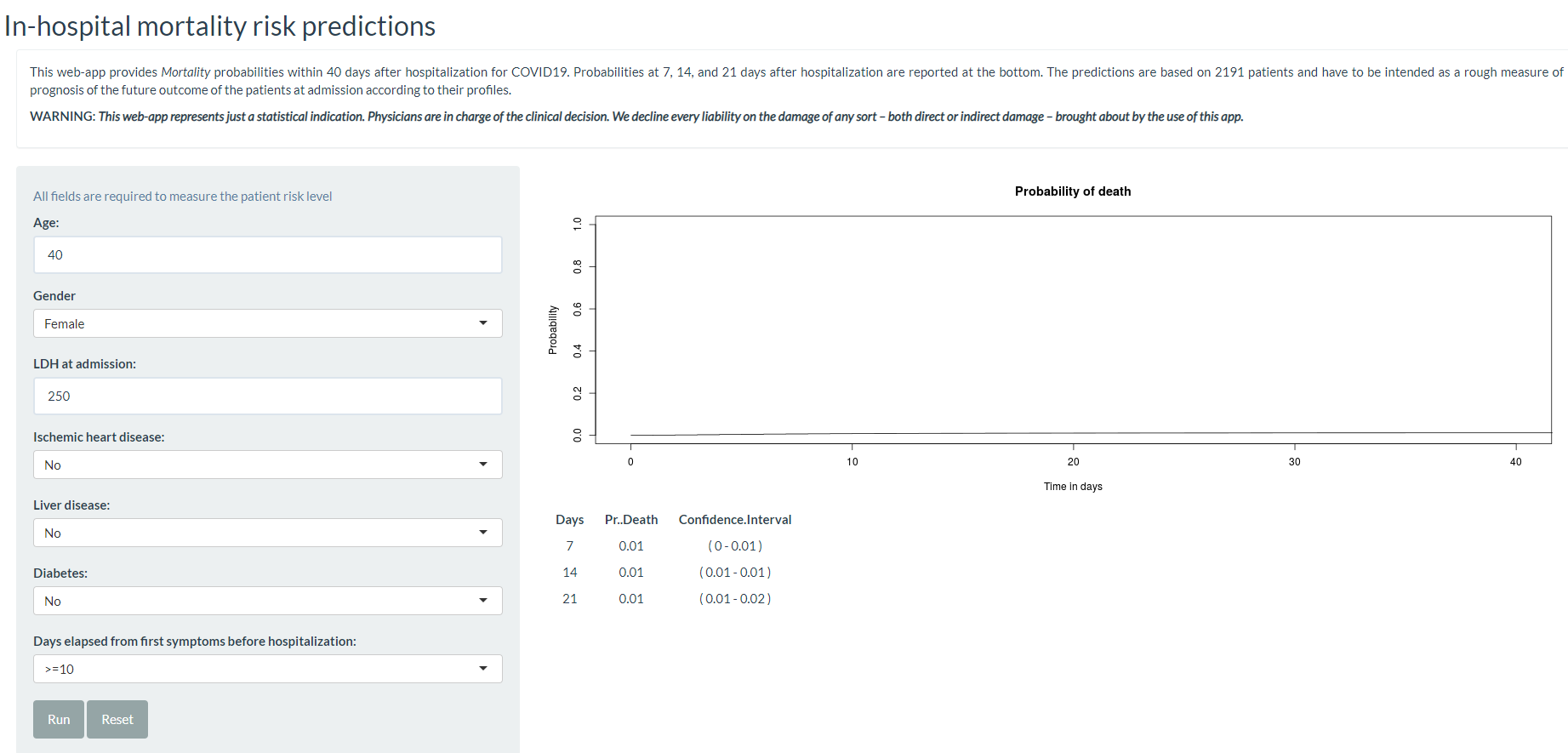

Supplement: S1 Fig — (TIF) [file pone.0245281.s002.tif]

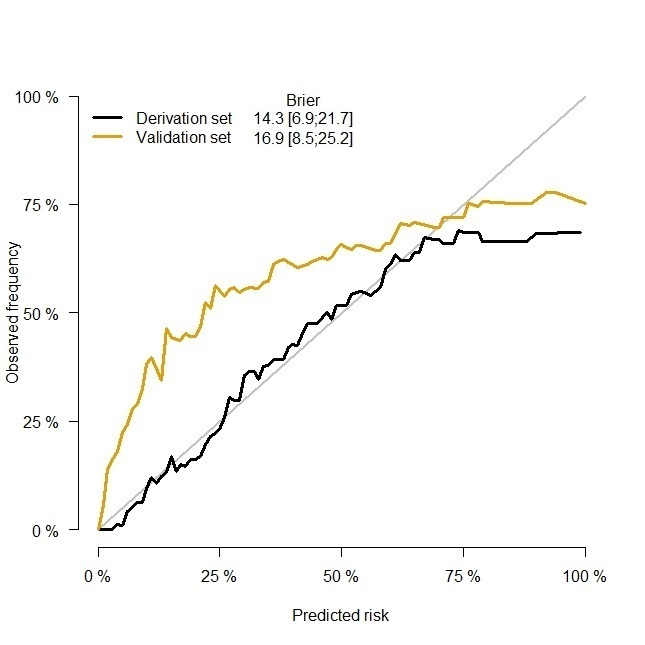

Supplement: S2 Fig — (TIF) [file pone.0245281.s003.tif]

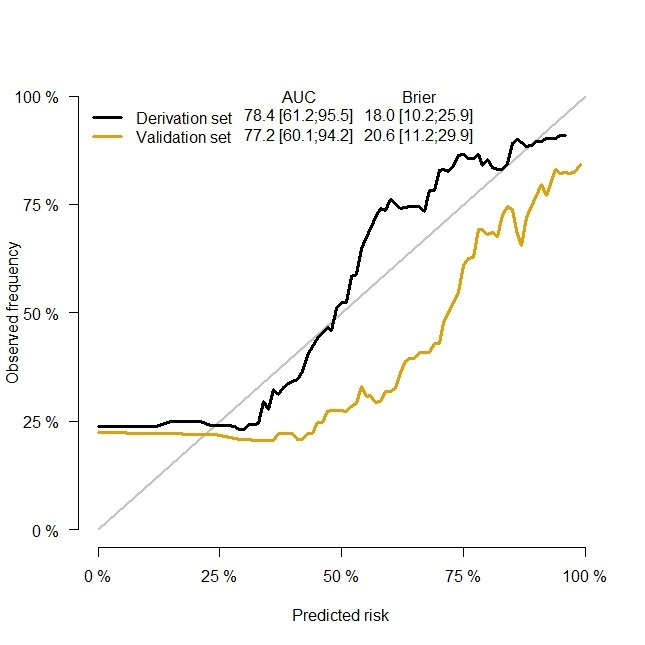

Supplement: S3 Fig — (TIF) [file pone.0245281.s004.tif]
