# Supplementary material for: Predicting in-hospital mortality from Coronavirus Disease 2019: A simple validated app for clinical use
Source: PLoS One. 2021 Jan 14;16(1):e0245281. doi: 10.1371/journal.pone.0245281 (PMC7808616; doi:10.1371/journal.pone.0245281)
Supplement: S1 File — (DOCX) [file pone.0245281.s005.docx]

**S1 File**

**Laboratory tests for SARS-COV2 diagnosis**

Total nucleic acids (DNA/RNA) were extracted from 200 ul of UTM™ using the QIAsymphon® instrument with QIAsymphony® DSP Virus/Pathogen Midi Kit (Complex 400 protocol) according to the manufacturer’s instructions (QIAGEN, Qiagen, Hilden, Germany). Specific RT-PCR targeting RNA-dependent RNA polymerase and E genes were used to detect the presence of SARS-CoV-2 in respiratory samples

**Supplementary Table S1.** Univariate analysis of risk factors for in-hospital mortality and discharge of patients with Coronavirus Disease-19 in the derivation cohort.

|  | Fine and Grey model | | | | | | | |
| --- | --- | --- | --- | --- | --- | --- | --- | --- |
|  | In-hospital mortality | | | | Discharge | | | |
|  | Beta | Standard Error | HR | p value | Beta | Standard Error | HR (95% CI) | p value |
| Age (years) | 0.07 | 0.003 | 1.08 | <0.0001 | -0.03 | 0.002 | 0.97 | <0.0001 |
| Male sex | 0.27 | 0.104 | 1.31 | 0.009 | -0.21 | 0.064 | 0.81 | 0.002 |
| Duration of symptoms before hospital admission shorter than 10 days | 0.63 | 0.106 | 1.85 | <0.0001 | -0.34 | 0.062 | 0.71 | <0.0001 |
| Number of comorbidities | 0.57 | 0.061 | 1.76 | <0.0001 | -0.49 | 0.057 | 0.62 | <0.0001 |
| Diabetes | 0.57 | 0.104 | 1.76 | <0.0001 | -0.49 | 0.085 | 0.61 | <0.0001 |
| Obesity | -0.02 | 0.139 | 0.98 | 0.903 | -0.12 | 0.081 | 0.88 | 0.130 |
| Coronary Heart Disease | 0.86 | 0.119 | 2.36 | <0.0001 | -0.66 | 0.130 | 0.51 | <0.0001 |
| Chronic obstructive lung disease | 0.45 | 0.167 | 1.58 | 0.107 | -0.19 | 0.131 | 0.83 | 0.146 |
| Malignancy | 0.46 | 0.199 | 1.59 | 0.120 | -0.29 | 0.176 | 0.75 | 0.102 |
| Chronic Liver disease | 0.74 | 0.209 | 2.09 | 0.0004 | -0.69 | 0.243 | 0.50 | 0.005 |
| Current smoker | -0.22 | 0.340 | 0.80 | 0.509 | 0.13 | 0.181 | 1.14 | 0.456 |
| Glutamic pyruvic transaminase (GPT) | -0.0001 | 0.001 | 1.00 | 0.915 | -0.0004 | 0.0004 | 1.00 | 0.317 |
| Lactate dehydrogenase (LDH) | 0.0004 | 0.0001 | 1.00 | <0.0001 | -0.002 | 0.0002 | 0.99 | <0.0001 |
| C-reactive protein (CRP)* | 0.02 | 0.004 | 1.02 | <0.0001 | -0.03 | 0.004 | 0.97 | <0.0001 |
| D-dimer * | 0.0001 | 0.0006 | 1.00 | 0.010 | -0.0001 | 0.0001 | 1.00 | 0.362 |
| White Cell Blood Count (WBC) | 0.02 | 0.012 | 1.02 | 0.211 | -0.03 | 0.009 | 0.97 | 0.001 |
| Lymphocyte Count* | -0.12 | 0.122 | 0.88 | 0.311 | 0.01 | 0.012 | 1.02 | 0.232 |
| Platelet Count | -0.02 | 0.006 | 0.98 | 0.0002 | 0.01 | 0.003 | 1.01 | 0.038 |
| P/F ratio * | -0.003 | 0.001 | 0.99 | 0.0003 | 0.002 | 0.0004 | 1.00 | <0.0001 |
| Dexamethasone | 0.03 | 0.17 | 1.03 | 0.870 | -0.36 | 0.70 | 0.12 | 0.003 |

*GPT, LDH, WBC, platelets, CRP, lymphocytes, D-dimer and P/F ratio were missing in 617 (34.0%), 211 (11.6%), 558 (30.8%), 248 (13.7%), 471 (26.0%), 852 (47.0%), 1592 (87.9%) and 884 patients (48.9%), respectively.

**Supplementary Table 2.** Risk factors for in-hospital mortality and discharge of patients with Coronavirus Disease-19 in the derivation cohort after inclusion of covariates with p-value <0.02 at univariate analysis.

|  | Derivation cohort | | | | | | | |
| --- | --- | --- | --- | --- | --- | --- | --- | --- |
|  | In-hospital mortality | | | | Discharge | | | |
|  | Beta | Standard Error | HR (95% CI) | p value | Beta | Standard Error | HR (95% CI) | p value |
| Age (years) | 0.08 | 0.004 | 1.08 (1.07-1.09) | <0.001 | -0.03 | 0.002 | 0.97 (0.96-0.98) | <0.001 |
| Male sex | 0.47 | 0.11 | 1.99 (1.29-1.99) | <0.001 | -0.21 | 0.07 | 0.81 (0.71-0.92) | <0.001 |
| Duration of symptoms before hospital admission shorter than 10 days | 0.53 | 0.11 | 1.70 (1.37-2.09) | <0.001 | -0.28 | 0.06 | 0.76 (0.67-0.86) | <0.001 |
| Type 2 diabetes | 0.20 | 0.11 | 1.22 (0.98-1.52) | 0.074 | -0.32 | 0.09 | 0.72 (0.61-0.86) | <0.001 |
| Coronary heart disease | 0.35 | 1.82 | 1.41 (1.10-1.82) | 0.007 | -0.33 | 0.13 | 0.72 (0.56-0.93) | 0.012 |
| Chronic liver disease | 0.49 | 0.23 | 1.63 (1.04-2.57) | 0.033 | -0.60 | 0.27 | 0.55 (0.32-0.93) | 0.026 |
| Chronic obstructive lung disease | -0.09 | 0.17 | 0.91 (0.65-1.27) | 0.580 | 0.20 | 0.14 | 1.22 (0.94-1.60) | 0.130 |
| Malignancy | 0.41 | 0.22 | 1.51 (0.97-2.34) | 0.067 | -0.19 | 0.17 | 0.83 (0.59-1.15) | 0.260 |
| Lactate dehydrogenase, U/L | 0.001 | 0.0001 | 1.0004 (1.0001-1.0005) | <0.001 | -0.001 | -0.002 | 0.998 (0.997-0.999) | <0.001 |
